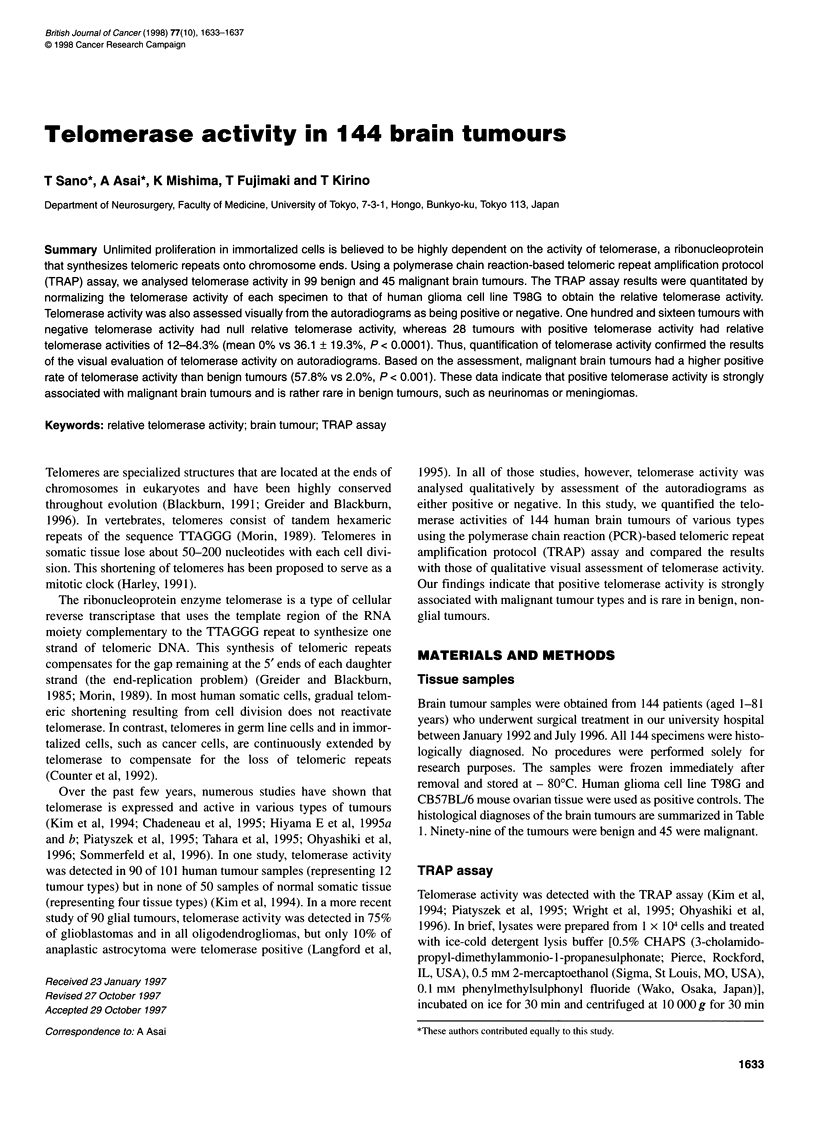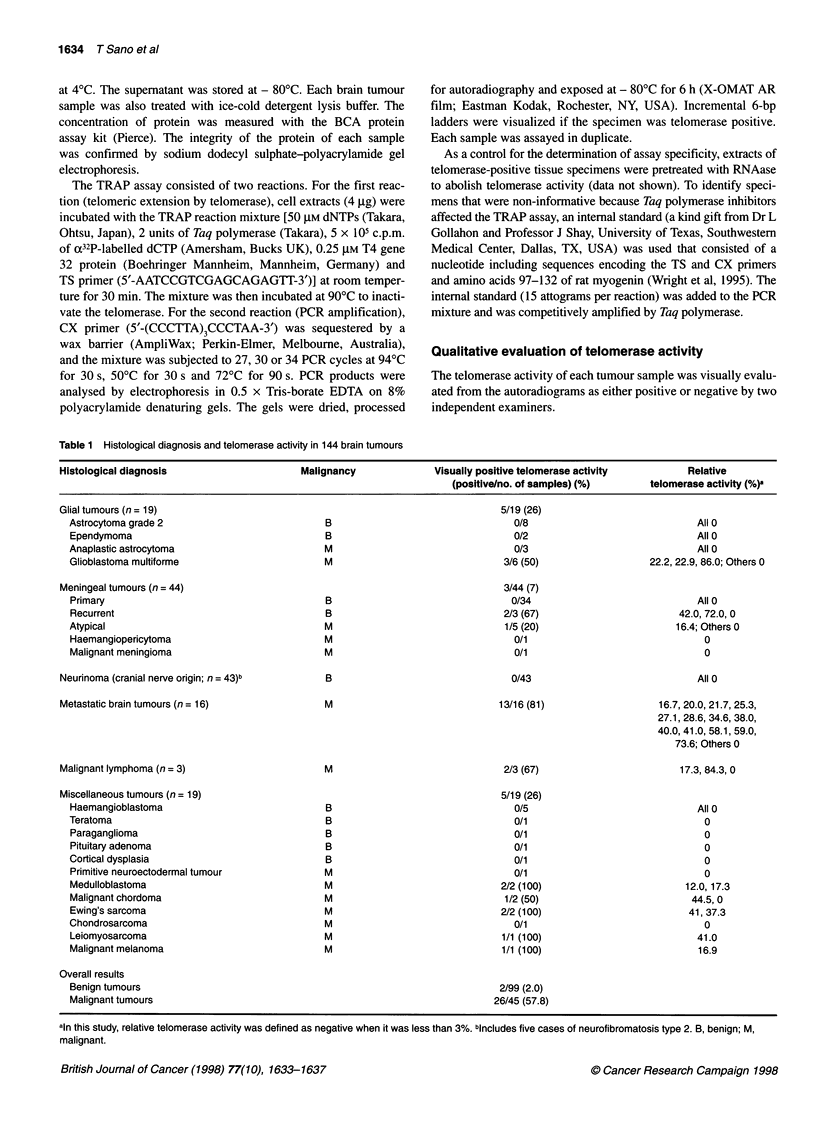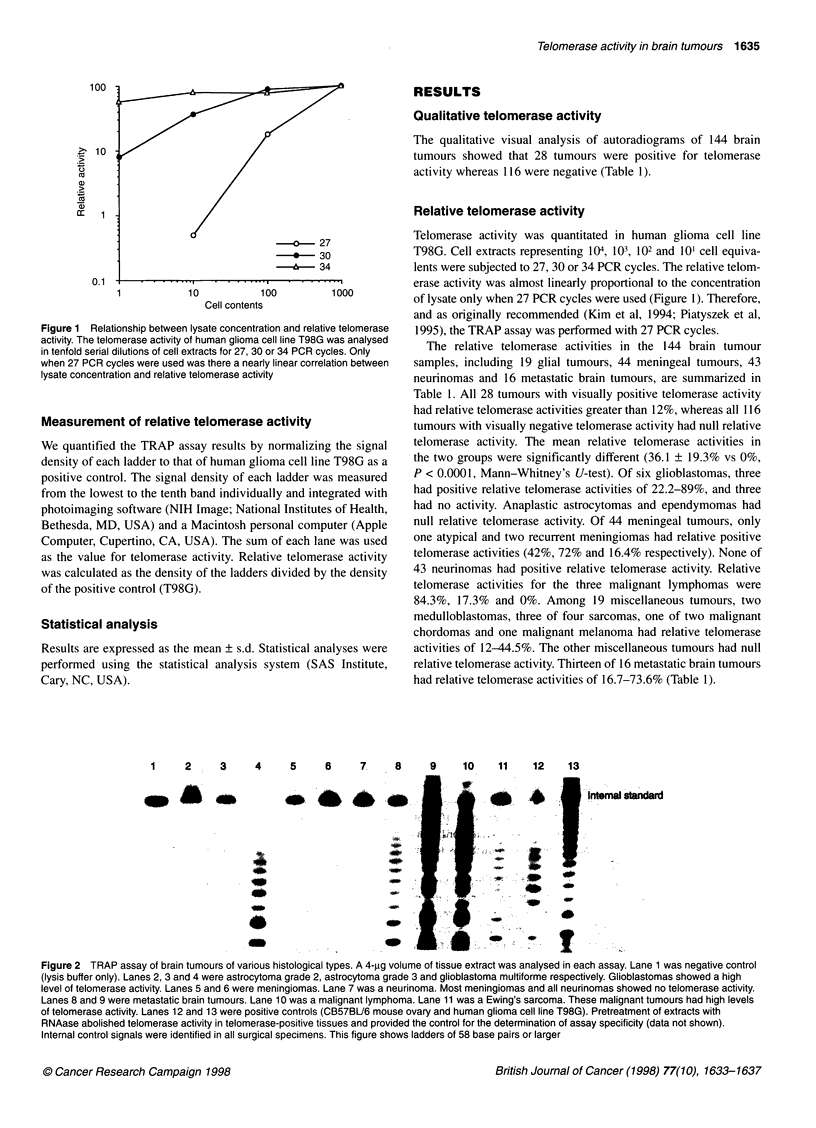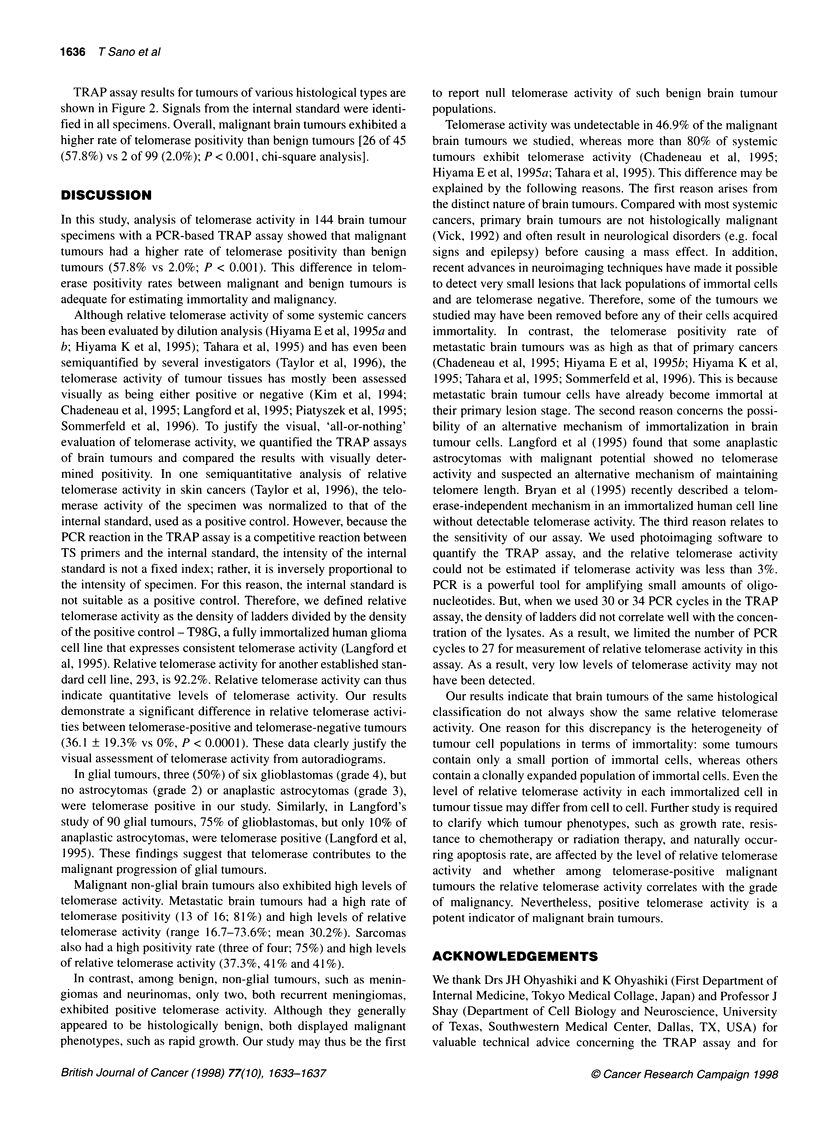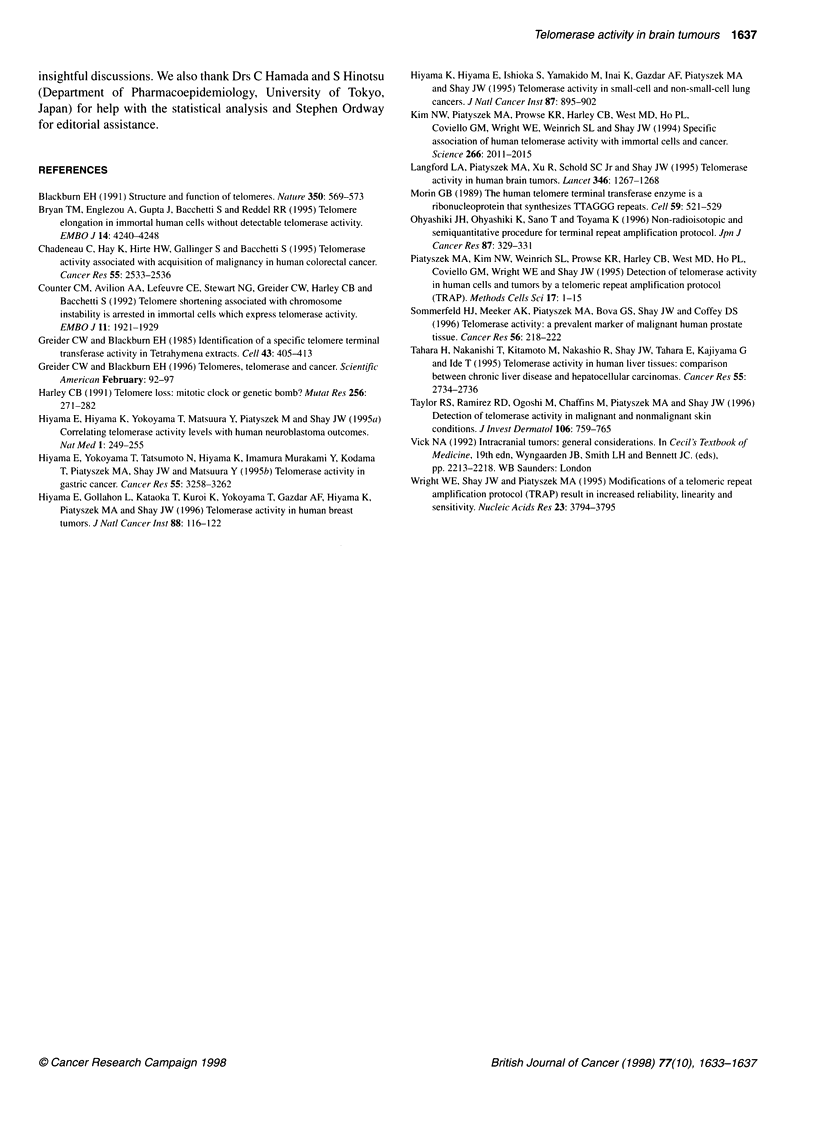# Telomerase activity in 144 brain tumours.

**DOI:** 10.1038/bjc.1998.267

**Published:** 1998-05

**Authors:** T. Sano, A. Asai, K. Mishima, T. Fujimaki, T. Kirino

**Affiliations:** Department of Neurosurgery, Faculty of Medicine, University of Tokyo, Japan.

## Abstract

**Images:**


*British Journal of Cancer* (1998) **77**(10), 1633–1637
© 1998 Cancer Research Campaign

### Telomerase activity in 144 brain tumours

**T Sano*, A Asai*, K Mishima, T Fujimaki and T Kirino**

Department of Neurosurgery, Faculty of Medicine, University of Tokyo, 7-3-1, Hongo, Bunkyo-ku, Tokyo 113, Japan

**Summary** Unlimited proliferation in immortalized cells is believed to be highly dependent on the activity of telomerase, a ribonucleoprotein that synthesizes telomeric repeats onto chromosome ends. Using a polymerase chain reaction-based telomeric repeat amplification protocol (TRAP) assay, we analysed telomerase activity in 99 benign and 45 malignant brain tumours. The TRAP assay results were quantitated by normalizing the telomerase activity of each specimen to that of human glioma cell line T98G to obtain the relative telomerase activity. Telomerase activity was also assessed visually from the autoradiograms as being positive or negative. One hundred and sixteen tumours with negative telomerase activity had null relative telomerase activity, whereas 28 tumours with positive telomerase activity had relative telomerase activities of 12–84.3% (mean 0% vs 36.1 ± 19.3%, $P < 0.0001$). Thus, quantification of telomerase activity confirmed the results of the visual evaluation of telomerase activity on autoradiograms. Based on the assessment, malignant brain tumours had a higher positive rate of telomerase activity than benign tumours (57.8% vs 2.0%, $P < 0.001$). These data indicate that positive telomerase activity is strongly associated with malignant brain tumours and is rather rare in benign tumours, such as neurinomas or meningiomas.

**Keywords:** relative telomerase activity; brain tumour; TRAP assay

Telomeres are specialized structures that are located at the ends of chromosomes in eukaryotes and have been highly conserved throughout evolution (Blackburn, 1991; Greider and Blackburn, 1996). In vertebrates, telomeres consist of tandem hexameric repeats of the sequence TTAGGG (Morin, 1989). Telomeres in somatic tissue lose about 50–200 nucleotides with each cell division. This shortening of telomeres has been proposed to serve as a mitotic clock (Harley, 1991).

The ribonucleoprotein enzyme telomerase is a type of cellular reverse transcriptase that uses the template region of the RNA moiety complementary to the TTAGGG repeat to synthesize one strand of telomeric DNA. This synthesis of telomeric repeats compensates for the gap remaining at the 5′ ends of each daughter strand (the end-replication problem) (Greider and Blackburn, 1985; Morin, 1989). In most human somatic cells, gradual telomeric shortening resulting from cell division does not reactivate telomerase. In contrast, telomeres in germ line cells and in immortalized cells, such as cancer cells, are continuously extended by telomerase to compensate for the loss of telomeric repeats (Counter et al, 1992).

Over the past few years, numerous studies have shown that telomerase is expressed and active in various types of tumours (Kim et al, 1994; Chadeneau et al, 1995; Hiyama E et al, 1995*a* and *b*; Piatyszek et al, 1995; Tahara et al, 1995; Ohyashiki et al, 1996; Sommerfeld et al, 1996). In one study, telomerase activity was detected in 90 of 101 human tumour samples (representing 12 tumour types) but in none of 50 samples of normal somatic tissue (representing four tissue types) (Kim et al, 1994). In a more recent study of 90 glial tumours, telomerase activity was detected in 75% of glioblastomas and in all oligodendrogliomas, but only 10% of anaplastic astrocytoma were telomerase positive (Langford et al, 1995). In all of those studies, however, telomerase activity was analysed qualitatively by assessment of the autoradiograms as either positive or negative. In this study, we quantified the telomerase activities of 144 human brain tumours of various types using the polymerase chain reaction (PCR)-based telomeric repeat amplification protocol (TRAP) assay and compared the results with those of qualitative visual assessment of telomerase activity. Our findings indicate that positive telomerase activity is strongly associated with malignant tumour types and is rare in benign, non-glial tumours.

*Received 23 January 1997*
*Revised 27 October 1997*
*Accepted 29 October 1997*

*Correspondence to:* A Asai

### MATERIALS AND METHODS

### Tissue samples

Brain tumour samples were obtained from 144 patients (aged 1–81 years) who underwent surgical treatment in our university hospital between January 1992 and July 1996. All 144 specimens were histologically diagnosed. No procedures were performed solely for research purposes. The samples were frozen immediately after removal and stored at – 80°C. Human glioma cell line T98G and CB57BL/6 mouse ovarian tissue were used as positive controls. The histological diagnoses of the brain tumours are summarized in Table 1. Ninety-nine of the tumours were benign and 45 were malignant.

### TRAP assay

Telomerase activity was detected with the TRAP assay (Kim et al, 1994; Piatyszek et al, 1995; Wright et al, 1995; Ohyashiki et al, 1996). In brief, lysates were prepared from $1 \times 10^4$ cells and treated with ice-cold detergent lysis buffer [0.5% CHAPS (3-cholamidopropyl-dimethylammonio-1-propanesulphonate; Pierce, Rockford, IL, USA), 0.5 mM 2-mercaptoethanol (Sigma, St Louis, MO, USA), 0.1 mM phenylmethylsulphonyl fluoride (Wako, Osaka, Japan)], incubated on ice for 30 min and centrifuged at 10 000 ***g*** for 30 min

*These authors contributed equally to this study.

at 4°C. The supernatant was stored at – 80°C. Each brain tumour sample was also treated with ice-cold detergent lysis buffer. The concentration of protein was measured with the BCA protein assay kit (Pierce). The integrity of the protein of each sample was confirmed by sodium dodecyl sulphate–polyacrylamide gel electrophoresis.

The TRAP assay consisted of two reactions. For the first reaction (telomeric extension by telomerase), cell extracts (4 μg) were incubated with the TRAP reaction mixture [50 μM dNTPs (Takara, Ohtsu, Japan), 2 units of *Taq* polymerase (Takara), $5 \times 10^5$ c.p.m. of $\alpha^{32}$P-labelled dCTP (Amersham, Bucks UK), 0.25 μM T4 gene 32 protein (Boehringer Mannheim, Mannheim, Germany) and TS primer (5′-AATCCGTCGAGCAGAGTT-3′)] at room temperture for 30 min. The mixture was then incubated at 90°C to inactivate the telomerase. For the second reaction (PCR amplification), CX primer (5′-$(CCCTTA)_3$CCCTAA-3′) was sequestered by a wax barrier (AmpliWax; Perkin-Elmer, Melbourne, Australia), and the mixture was subjected to 27, 30 or 34 PCR cycles at 94°C for 30 s, 50°C for 30 s and 72°C for 90 s. PCR products were analysed by electrophoresis in 0.5 × Tris-borate EDTA on 8% polyacrylamide denaturing gels. The gels were dried, processed for autoradiography and exposed at – 80°C for 6 h (X-OMAT AR film; Eastman Kodak, Rochester, NY, USA). Incremental 6-bp ladders were visualized if the specimen was telomerase positive. Each sample was assayed in duplicate.

As a control for the determination of assay specificity, extracts of telomerase-positive tissue specimens were pretreated with RNAase to abolish telomerase activity (data not shown). To identify specimens that were non-informative because *Taq* polymerase inhibitors affected the TRAP assay, an internal standard (a kind gift from Dr L Gollahon and Professor J Shay, University of Texas, Southwestern Medical Center, Dallas, TX, USA) was used that consisted of a nucleotide including sequences encoding the TS and CX primers and amino acids 97–132 of rat myogenin (Wright et al, 1995). The internal standard (15 attograms per reaction) was added to the PCR mixture and was competitively amplified by *Taq* polymerase.

### Qualitative evaluation of telomerase activity

The telomerase activity of each tumour sample was visually evaluated from the autoradiograms as either positive or negative by two independent examiners.

**Table 1** Histological diagnosis and telomerase activity in 144 brain tumours

| Histological diagnosis | Malignancy | Visually positive telomerase activity (positive/no. of samples) (%) | Relative telomerase activity (%)[a] |
|---|---|---|---|
| Glial tumours (*n* = 19) | | 5/19 (26) | |
| Astrocytoma grade 2 | B | 0/8 | All 0 |
| Ependymoma | B | 0/2 | All 0 |
| Anaplastic astrocytoma | M | 0/3 | All 0 |
| Glioblastoma multiforme | M | 3/6 (50) | 22.2, 22.9, 86.0; Others 0 |
| Meningeal tumours (*n* = 44) | | 3/44 (7) | |
| Primary | B | 0/34 | All 0 |
| Recurrent | B | 2/3 (67) | 42.0, 72.0, 0 |
| Atypical | M | 1/5 (20) | 16.4; Others 0 |
| Haemangiopericytoma | M | 0/1 | 0 |
| Malignant meningioma | M | 0/1 | 0 |
| Neurinoma (cranial nerve origin; *n* = 43)[b] | B | 0/43 | All 0 |
| Metastatic brain tumours (*n* = 16) | M | 13/16 (81) | 16.7, 20.0, 21.7, 25.3, 27.1, 28.6, 34.6, 38.0, 40.0, 41.0, 58.1, 59.0, 73.6; Others 0 |
| Malignant lymphoma (*n* = 3) | M | 2/3 (67) | 17.3, 84.3, 0 |
| Miscellaneous tumours (*n* = 19) | | 5/19 (26) | |
| Haemangioblastoma | B | 0/5 | All 0 |
| Teratoma | B | 0/1 | 0 |
| Paraganglioma | B | 0/1 | 0 |
| Pituitary adenoma | B | 0/1 | 0 |
| Cortical dysplasia | B | 0/1 | 0 |
| Primitive neuroectodermal tumour | M | 0/1 | 0 |
| Medulloblastoma | M | 2/2 (100) | 12.0, 17.3 |
| Malignant chordoma | M | 1/2 (50) | 44.5, 0 |
| Ewing's sarcoma | M | 2/2 (100) | 41, 37.3 |
| Chondrosarcoma | M | 0/1 | 0 |
| Leiomyosarcoma | M | 1/1 (100) | 41.0 |
| Malignant melanoma | M | 1/1 (100) | 16.9 |
| Overall results | | | |
| Benign tumours | | 2/99 (2.0) | |
| Malignant tumours | | 26/45 (57.8) | |

[a]In this study, relative telomerase activity was defined as negative when it was less than 3%. [b]Includes five cases of neurofibromatosis type 2. B, benign; M, malignant.

*© Cancer Research Campaign 1998*

**Figure 1** Relationship between lysate concentration and relative telomerase activity. The telomerase activity of human glioma cell line T98G was analysed in tenfold serial dilutions of cell extracts for 27, 30 or 34 PCR cycles. Only when 27 PCR cycles were used was there a nearly linear correlation between lysate concentration and relative telomerase activity

### Measurement of relative telomerase activity

We quantified the TRAP assay results by normalizing the signal density of each ladder to that of human glioma cell line T98G as a positive control. The signal density of each ladder was measured from the lowest to the tenth band individually and integrated with photoimaging software (NIH Image; National Institutes of Health, Bethesda, MD, USA) and a Macintosh personal computer (Apple Computer, Cupertino, CA, USA). The sum of each lane was used as the value for telomerase activity. Relative telomerase activity was calculated as the density of the ladders divided by the density of the positive control (T98G).

### Statistical analysis

Results are expressed as the mean ± s.d. Statistical analyses were performed using the statistical analysis system (SAS Institute, Cary, NC, USA).

### RESULTS

### Qualitative telomerase activity

The qualitative visual analysis of autoradiograms of 144 brain tumours showed that 28 tumours were positive for telomerase activity whereas 116 were negative (Table 1).

### Relative telomerase activity

Telomerase activity was quantitated in human glioma cell line T98G. Cell extracts representing $10^4$, $10^3$, $10^2$ and $10^1$ cell equivalents were subjected to 27, 30 or 34 PCR cycles. The relative telomerase activity was almost linearly proportional to the concentration of lysate only when 27 PCR cycles were used (Figure 1). Therefore, and as originally recommended (Kim et al, 1994; Piatyszek et al, 1995), the TRAP assay was performed with 27 PCR cycles.

The relative telomerase activities in the 144 brain tumour samples, including 19 glial tumours, 44 meningeal tumours, 43 neurinomas and 16 metastatic brain tumours, are summarized in Table 1. All 28 tumours with visually positive telomerase activity had relative telomerase activities greater than 12%, whereas all 116 tumours with visually negative telomerase activity had null relative telomerase activity. The mean relative telomerase activities in the two groups were significantly different (36.1 ± 19.3% vs 0%, $P < 0.0001$, Mann–Whitney's $U$-test). Of six glioblastomas, three had positive relative telomerase activities of 22.2–89%, and three had no activity. Anaplastic astrocytomas and ependymomas had null relative telomerase activity. Of 44 meningeal tumours, only one atypical and two recurrent meningiomas had relative positive telomerase activities (42%, 72% and 16.4% respectively). None of 43 neurinomas had positive relative telomerase activity. Relative telomerase activities for the three malignant lymphomas were 84.3%, 17.3% and 0%. Among 19 miscellaneous tumours, two medulloblastomas, three of four sarcomas, one of two malignant chordomas and one malignant melanoma had relative telomerase activities of 12–44.5%. The other miscellaneous tumours had null relative telomerase activity. Thirteen of 16 metastatic brain tumours had relative telomerase activities of 16.7–73.6% (Table 1).

**Figure 2** TRAP assay of brain tumours of various histological types. A 4-μg volume of tissue extract was analysed in each assay. Lane 1 was negative control (lysis buffer only). Lanes 2, 3 and 4 were astrocytoma grade 2, astrocytoma grade 3 and glioblastoma multiforme respectively. Glioblastomas showed a high level of telomerase activity. Lanes 5 and 6 were meningiomas. Lane 7 was a neurinoma. Most meningiomas and all neurinomas showed no telomerase activity. Lanes 8 and 9 were metastatic brain tumours. Lane 10 was a malignant lymphoma. Lane 11 was a Ewing's sarcoma. These malignant tumours had high levels of telomerase activity. Lanes 12 and 13 were positive controls (CB57BL/6 mouse ovary and human glioma cell line T98G). Pretreatment of extracts with RNAase abolished telomerase activity in telomerase-positive tissues and provided the control for the determination of assay specificity (data not shown). Internal control signals were identified in all surgical specimens. This figure shows ladders of 58 base pairs or larger

© Cancer Research Campaign 1998

TRAP assay results for tumours of various histological types are shown in Figure 2. Signals from the internal standard were identified in all specimens. Overall, malignant brain tumours exhibited a higher rate of telomerase positivity than benign tumours [26 of 45 (57.8%) vs 2 of 99 (2.0%); $P < 0.001$, chi-square analysis].

### DISCUSSION

In this study, analysis of telomerase activity in 144 brain tumour specimens with a PCR-based TRAP assay showed that malignant tumours had a higher rate of telomerase positivity than benign tumours (57.8% vs 2.0%; $P < 0.001$). This difference in telomerase positivity rates between malignant and benign tumours is adequate for estimating immortality and malignancy.

Although relative telomerase activity of some systemic cancers has been evaluated by dilution analysis (Hiyama E et al, 1995*a* and *b*; Hiyama K et al, 1995); Tahara et al, 1995) and has even been semiquantified by several investigators (Taylor et al, 1996), the telomerase activity of tumour tissues has mostly been assessed visually as being either positive or negative (Kim et al, 1994; Chadeneau et al, 1995; Langford et al, 1995; Piatyszek et al, 1995; Sommerfeld et al, 1996). To justify the visual, 'all-or-nothing' evaluation of telomerase activity, we quantified the TRAP assays of brain tumours and compared the results with visually determined positivity. In one semiquantitative analysis of relative telomerase activity in skin cancers (Taylor et al, 1996), the telomerase activity of the specimen was normalized to that of the internal standard, used as a positive control. However, because the PCR reaction in the TRAP assay is a competitive reaction between TS primers and the internal standard, the intensity of the internal standard is not a fixed index; rather, it is inversely proportional to the intensity of specimen. For this reason, the internal standard is not suitable as a positive control. Therefore, we defined relative telomerase activity as the density of ladders divided by the density of the positive control – T98G, a fully immortalized human glioma cell line that expresses consistent telomerase activity (Langford et al, 1995). Relative telomerase activity for another established standard cell line, 293, is 92.2%. Relative telomerase activity can thus indicate quantitative levels of telomerase activity. Our results demonstrate a significant difference in relative telomerase activities between telomerase-positive and telomerase-negative tumours (36.1 ± 19.3% vs 0%, $P < 0.0001$). These data clearly justify the visual assessment of telomerase activity from autoradiograms.

In glial tumours, three (50%) of six glioblastomas (grade 4), but no astrocytomas (grade 2) or anaplastic astrocytomas (grade 3), were telomerase positive in our study. Similarly, in Langford's study of 90 glial tumours, 75% of glioblastomas, but only 10% of anaplastic astrocytomas, were telomerase positive (Langford et al, 1995). These findings suggest that telomerase contributes to the malignant progression of glial tumours.

Malignant non-glial brain tumours also exhibited high levels of telomerase activity. Metastatic brain tumours had a high rate of telomerase positivity (13 of 16; 81%) and high levels of relative telomerase activity (range 16.7–73.6%; mean 30.2%). Sarcomas also had a high positivity rate (three of four; 75%) and high levels of relative telomerase activity (37.3%, 41% and 41%).

In contrast, among benign, non-glial tumours, such as meningiomas and neurinomas, only two, both recurrent meningiomas, exhibited positive telomerase activity. Although they generally appeared to be histologically benign, both displayed malignant phenotypes, such as rapid growth. Our study may thus be the first to report null telomerase activity of such benign brain tumour populations.

Telomerase activity was undetectable in 46.9% of the malignant brain tumours we studied, whereas more than 80% of systemic tumours exhibit telomerase activity (Chadeneau et al, 1995; Hiyama E et al, 1995*a*; Tahara et al, 1995). This difference may be explained by the following reasons. The first reason arises from the distinct nature of brain tumours. Compared with most systemic cancers, primary brain tumours are not histologically malignant (Vick, 1992) and often result in neurological disorders (e.g. focal signs and epilepsy) before causing a mass effect. In addition, recent advances in neuroimaging techniques have made it possible to detect very small lesions that lack populations of immortal cells and are telomerase negative. Therefore, some of the tumours we studied may have been removed before any of their cells acquired immortality. In contrast, the telomerase positivity rate of metastatic brain tumours was as high as that of primary cancers (Chadeneau et al, 1995; Hiyama E et al, 1995*b*; Hiyama K et al, 1995; Tahara et al, 1995; Sommerfeld et al, 1996). This is because metastatic brain tumour cells have already become immortal at their primary lesion stage. The second reason concerns the possibility of an alternative mechanism of immortalization in brain tumour cells. Langford et al (1995) found that some anaplastic astrocytomas with malignant potential showed no telomerase activity and suspected an alternative mechanism of maintaining telomere length. Bryan et al (1995) recently described a telomerase-independent mechanism in an immortalized human cell line without detectable telomerase activity. The third reason relates to the sensitivity of our assay. We used photoimaging software to quantify the TRAP assay, and the relative telomerase activity could not be estimated if telomerase activity was less than 3%. PCR is a powerful tool for amplifying small amounts of oligonucleotides. But, when we used 30 or 34 PCR cycles in the TRAP assay, the density of ladders did not correlate well with the concentration of the lysates. As a result, we limited the number of PCR cycles to 27 for measurement of relative telomerase activity in this assay. As a result, very low levels of telomerase activity may not have been detected.

Our results indicate that brain tumours of the same histological classification do not always show the same relative telomerase activity. One reason for this discrepancy is the heterogeneity of tumour cell populations in terms of immortality: some tumours contain only a small portion of immortal cells, whereas others contain a clonally expanded population of immortal cells. Even the level of relative telomerase activity in each immortalized cell in tumour tissue may differ from cell to cell. Further study is required to clarify which tumour phenotypes, such as growth rate, resistance to chemotherapy or radiation therapy, and naturally occurring apoptosis rate, are affected by the level of relative telomerase activity and whether among telomerase-positive malignant tumours the relative telomerase activity correlates with the grade of malignancy. Nevertheless, positive telomerase activity is a potent indicator of malignant brain tumours.

### ACKNOWLEDGEMENTS

We thank Drs JH Ohyashiki and K Ohyashiki (First Department of Internal Medicine, Tokyo Medical Collage, Japan) and Professor J Shay (Department of Cell Biology and Neuroscience, University of Texas, Southwestern Medical Center, Dallas, TX, USA) for valuable technical advice concerning the TRAP assay and for

© Cancer Research Campaign 1998

insightful discussions. We also thank Drs C Hamada and S Hinotsu (Department of Pharmacoepidemiology, University of Tokyo, Japan) for help with the statistical analysis and Stephen Ordway for editorial assistance.

### REFERENCES

Blackburn EH (1991) Structure and function of telomeres. *Nature* **350**: 569–573

Bryan TM, Englezou A, Gupta J, Bacchetti S and Reddel RR (1995) Telomere elongation in immortal human cells without detectable telomerase activity. *EMBO J* **14**: 4240–4248

Chadeneau C, Hay K, Hirte HW, Gallinger S and Bacchetti S (1995) Telomerase activity associated with acquisition of malignancy in human colorectal cancer. *Cancer Res* **55**: 2533–2536

Counter CM, Avilion AA, Lefeuvre CE, Stewart NG, Greider CW, Harley CB and Bacchetti S (1992) Telomere shortening associated with chromosome instability is arrested in immortal cells which express telomerase activity. *EMBO J* **11**: 1921–1929

Greider CW and Blackburn EH (1985) Identification of a specific telomere terminal transferase activity in Tetrahymena extracts. *Cell* **43**: 405–413

Greider CW and Blackburn EH (1996) Telomeres, telomerase and cancer. *Scientific American* **February**: 92–97

Harley CB (1991) Telomere loss: mitotic clock or genetic bomb? *Mutat Res* **256**: 271–282

Hiyama E, Hiyama K, Yokoyama T, Matsuura Y, Piatyszek M and Shay JW (1995*a*) Correlating telomerase activity levels with human neuroblastoma outcomes. *Nat Med* **1**: 249–255

Hiyama E, Yokoyama T, Tatsumoto N, Hiyama K, Imamura Murakami Y, Kodama T, Piatyszek MA, Shay JW and Matsuura Y (1995*b*) Telomerase activity in gastric cancer. *Cancer Res* **55**: 3258–3262

Hiyama E, Gollahon L, Kataoka T, Kuroi K, Yokoyama T, Gazdar AF, Hiyama K, Piatyszek MA and Shay JW (1996) Telomerase activity in human breast tumors. *J Natl Cancer Inst* **88**: 116–122

Hiyama K, Hiyama E, Ishioka S, Yamakido M, Inai K, Gazdar AF, Piatyszek MA and Shay JW (1995) Telomerase activity in small-cell and non-small-cell lung cancers. *J Natl Cancer Inst* **87**: 895–902

Kim NW, Piatyszek MA, Prowse KR, Harley CB, West MD, Ho PL. Coviello GM, Wright WE, Weinrich SL and Shay JW (1994) Specific association of human telomerase activity with immortal cells and cancer. *Science* **266**: 2011–2015

Langford LA, Piatyszek MA, Xu R, Schold SC Jr and Shay JW (1995) Telomerase activity in human brain tumors. *Lancet* **346**: 1267–1268

Morin GB (1989) The human telomere terminal transferase enzyme is a ribonucleoprotein that synthesizes TTAGGG repeats. *Cell* **59**: 521–529

Ohyashiki JH, Ohyashiki K, Sano T and Toyama K (1996) Non-radioisotopic and semiquantitative procedure for terminal repeat amplification protocol. *Jpn J Cancer Res* **87**: 329–331

Piatyszek MA, Kim NW, Weinrich SL, Prowse KR, Harley CB, West MD, Ho PL, Coviello GM, Wright WE and Shay JW (1995) Detection of telomerase activity in human cells and tumors by a telomeric repeat amplification protocol (TRAP). *Methods Cells Sci* **17**: 1–15

Sommerfeld HJ, Meeker AK, Piatyszek MA, Bova GS, Shay JW and Coffey DS (1996) Telomerase activity: a prevalent marker of malignant human prostate tissue. *Cancer Res* **56**: 218–222

Tahara H, Nakanishi T, Kitamoto M, Nakashio R, Shay JW, Tahara E, Kajiyama G and Ide T (1995) Telomerase activity in human liver tissues: comparison between chronic liver disease and hepatocellular carcinomas. *Cancer Res* **55**: 2734–2736

Taylor RS, Ramirez RD, Ogoshi M, Chaffins M, Piatyszek MA and Shay JW (1996) Detection of telomerase activity in malignant and nonmalignant skin conditions. *J Invest Dermatol* **106**: 759–765

Vick NA (1992) Intracranial tumors: general considerations. In *Cecil's Textbook of Medicine*, 19th edn, Wyngaarden JB, Smith LH and Bennett JC. (eds), pp. 2213–2218. WB Saunders: London

Wright WE, Shay JW and Piatyszek MA (1995) Modifications of a telomeric repeat amplification protocol (TRAP) result in increased reliability, linearity and sensitivity. *Nucleic Acids Res* **23**: 3794–3795

*© Cancer Research Campaign 1998*